# Language Impairment in Adolescents with Sydenham Chorea

**DOI:** 10.15844/pedneurbriefs-29-11-6

**Published:** 2015-11

**Authors:** J. Gordon Millichap

**Affiliations:** 1 Division of Neurology, Ann & Robert H. Lurie Children's Hospital of Chicago, Chicago, IL; 2 Departments of Pediatrics and Neurology, Northwestern University Feinberg School of Medicine, Chicago, IL

**Keywords:** Sydenham Chorea, Rheumatic Fever, Verbal Fluency, Comprehension

## Abstract

Investigators from hospitals in Brazil tested verbal fluency in 20 adolescent patients, ages ranged from 11 to 16 years (mean 13.8 years), with Sydenham chorea compared with 20 patients with rheumatic fever without chorea and 20 healthy controls, matched for age and gender.

Investigators from hospitals in Brazil tested verbal fluency in 20 adolescent patients, ages ranged from 11 to 16 years (mean 13.8 years), with Sydenham chorea compared with 20 patients with rheumatic fever without chorea and 20 healthy controls, matched for age and gender. Performance in verbal fluency and in verbal comprehension tasks differed significantly (P < 0.01) among the three groups. Patients with Sydenham chorea performed significantly worse than healthy control group in phonemic and semantic verbal fluency tasks as well as verbal comprehension (Token Test). The rheumatic fever group also performed worse than healthy controls in phonemic verbal fluency. Severity of motor signs in Sydenham chorea correlated inversely with performance in phonemic verbal fluency. [1]

COMMENTARY. Nineteenth century reports of clinical manifestations of Sydenham or rheumatic chorea refer to speech but not language problems. Cheadle WB of the Great Ormond Street Hospital, London, UK, describes explosive bursts of inarticulate speech, jerky, or Sydenham's speech [2]. The choreiform movements involving the face, tongue, palate and larynx result in severe dysarthria, stammer and hesitation in talking. The present study using tasks of verbal fluency and verbal comprehension finds an association of rheumatic chorea with receptive and expressive language impairment [1].

The incidence of Sydenham chorea and acute rheumatic fever has declined dramatically in recent years but the disease and neuropsychiatric complications still cause significant morbidity in developing countries. In a follow-up study of 65 Sydenham chorea patients in Turkey, mean age at onset of symptoms was 11.7 +/-2.6 years (range 6-17 years), and 63% were female. History of rheumatic fever was recorded in 30.8% of patients, carditis and EKG valve involvement in 70.5%, normal brain MRI in all of 18 tested, and abnormal slow waves in the EEG of 50% of 18 tested. Recovery following the first attack of chorea occurred in 1 to 6 months in 51.7% of patients, and recurrence in 37.9% [3].

The motor features of rheumatic chorea are often complicated by neuropsychiatric disorders, most commonly obsessive-compulsive behavior, and less frequently ADHD, anxiety, and depression [4]. The prevalence of ADHD before and after chorea was 30% and 37%, respectively. The prevalence for anxiety before, during, and after Sydenham's chorea was 71, 79, and 79%, and for depression, 19, 69, and 44%. Streptococcal antibody titers and duration of treatment did not correlate with ADHD, depression, or anxiety disorders. Seizures are reported rarely with Sydenham chorea, but reports of the EEG are abnormal in ∼50%, returning to normal in one to 4 weeks [5, 6].
